# Ageing behavior of extruded Mg–8.2Gd–3.8Y–1.0Zn–0.4Zr (wt.%) alloy containing LPSO phase and γ′ precipitates

**DOI:** 10.1038/srep43391

**Published:** 2017-02-23

**Authors:** C. Xu, T. Nakata, X. G. Qiao, M. Y. Zheng, K. Wu, S. Kamado

**Affiliations:** 1 School of Materials Science and Engineering, Harbin Institute of Technology, Harbin 150001, PR China; 2 Research Center for Advanced Magnesium Technology, Nagaoka University of Technology, Nagaoka 940-2188, Japan

## Abstract

The effect of long period stacking ordered (LPSO) phase and γ′ precipitates on the ageing behavior and mechanical properties of the extruded Mg–8.2Gd–3.8Y–1.0Zn–0.4Zr (wt.%) alloy was investigated. The results show that more β′ phases precipitate during ageing treatment in the LPSO phase containing alloy so that the LPSO phase containing alloy exhibits a higher age-hardening response than the γ′ precipitates containing alloy. The precipitation strengthening induced by β′ precipitates is the greatest contributor to the strength of the peak-aged LPSO-containing alloys. Higher strength is achieved in γ′ precipitates containing alloy due to the more effective strengthening induced by dense nanoscale γ′ precipitates than LPSO phases as well as the higher volume fraction of coarse unrecrystallized grains with strong basal texture. The extruded alloy containing γ′ precipitates after T5 peak-ageing treatment shows ultra-high tensile yield strength of 462 MPa, high ultimate tensile strength of 520 MPa, and superior elongation to failure of 10.6%.

In recent decades, growing attention has been paid to the weight reduction in vehicle components to achieve high fuel efficiency and reduce the CO_2_ emission. As the lightest metallic structural materials, Mg alloys exhibit low density, high specific strength and good damping capacities, having great potential in automotive and aerospace applications^1^,^2^,^3^,^4^. However, their low strength, especially at elevated temperatures, is the major obstacle for their wider commercial applications. The addition of heavy rare earth (HRE) elements, such as Gd and Y, into Mg alloys leads to obvious age-hardening response and remarkably improved strength, which is caused by the precipitation of metastable β′ phase on the prismatic planes of α-Mg matrix^5^. However, these strengthening phases deteriorate the ductility so that the elongation to failure decreases obviously after ageing treatment^6^.

Mg–Gd binary alloys with Gd contents less than 10 wt.% exhibit negligible precipitation hardening effect^7^. However, 1~2 wt.% Zn addition to Mg–6Gd–0.6Zr (wt.%) alloy remarkably enhance the age hardening response by the formation of dense basal precipitate plates^8^. Furthermore, Zn addition to the Mg–HRE alloys induces the formation of a novel phase with long period stacking ordered (LPSO) structure and/or the solute segregated stacking faults (SFs) on the basal planes of α-Mg matrix^5^,^8^,^9^,^10^,^11^,^12^,^13^,^14^,^15^,^16^,^17^. According to the analysis of the precipitation behavior of Mg–Gd–Zn–Zr alloys^8^,^10^, these so-called solute segregated SFs are demonstrated to be the precipitates on basal planes named γ′ precipitates. The LPSO phase containing nanocrystalline Mg_2_Y_1_Zn (at.%) alloy processed by rapid solidification powder metallurgy exhibits ultrahigh tensile yield strength of ~600 MPa at room temperature (RT)^13^. The LPSO phase with higher hardness than α-Mg matrix not only increases the strength but also contribute to the ductility of the Mg–Y–Zn alloy^14^. The γ′ precipitates are reported to be more beneficial for the strength improvement than LPSO phase in Mg–6.5Gd–2.5Dy–1.8Zn (wt.%) alloy^15^. In addition to the LPSO phases and/or γ′ precipitates on the basal planes, the metastable β′ phase precipitated on the prismatic plane plays an important role in strengthening of Mg–RE alloys. For the Mg-RE-Zn alloy system, when the rare-earth element is less than 10 wt.%, only LPSO phase/γ′ precipitates are formed through specific heat treatment^17^. However, the alloys containing rare earth elements more than 10 wt.% additionally have beta series phases which include β′, β_1_ and β phases^18^. In addition, based on the time-temperature-transformation (TTT) diagram of the precipitation of β series phases and LPSO phases and the formation of γ′ precipitates in Mg–Zn–Y alloy^9^, these phases are selectively formed by controlling the aging temperature and time. Only β′ phases are formed under low temperature aging treatment, while high temperature aging treatment induces the formation of LPSO and γ′ precipitates. Therefore the dominant strengthening mechanism would be dependent on the precipitate phase formed in the alloys and it is expected that high strength can be achieved by simultaneous precipitation of plate-shaped LPSO phases and/or γ′ precipitates on basal and β′ phase on prismatic planes of α-Mg alloys. Unfortunately, the effect of LPSO phase or γ′ precipitates on the precipitation behavior of β′ phases during ageing treatment of Mg–RE–Zn alloys has been rarely reported.

In this study, as-extruded Mg–8.2Gd–3.8Y–1.0Zn–0.4Zr (wt.%) alloy containing thin plate-shaped LPSO phase or dense fine γ′ precipitates was subjected to ageing treatment at 200 °C. The effect of these LPSO phases or γ′ precipitates on the age hardening response was investigated and high performance Mg alloys with simultaneous precipitates on basal and prismatic planes were obtained.

## Results

### Microstructure of the as-extruded alloy

**Figure 1:**
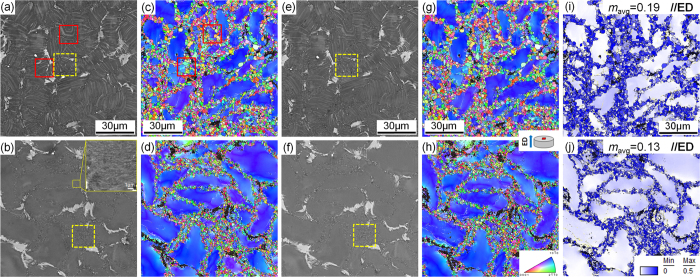
Quasi *in-situ* SEM observations and EBSD analysis of the extruded and peak-aged samples: (**a**,**b**,**e**,**f**) SEM micrographs, (**c**,**d**,**g**,**h**) IPF maps and (**i**,**j**) (0001) <11
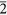
0> Schmid factor distribution maps; (**a**,**c**) FE, (**b**,**d**) QE, (**e**,**g**,**i**) FEA, (**f**,**h**,**j**) QEA.

Figure 1a and b shows the SEM micrographs observed along extrusion directions (ED) of the FE and QE samples, respectively. The block-shaped phases are deformed and bimodal microstructure comprising coarse unrecrystallized (unDRXed) grains and fine dynamically recrystallized (DRXed) grains is observed in both extruded alloys. Plate-shaped LPSO phases in the furnace-cooled sample retain after extrusion processing, some kink deformation can be observed in the LPSO phases, as shown in Fig. 1a. According to the inverse pole figure (IPF) maps of the extruded alloys shown in Fig. 1c and d, the coarse unDRXed grains mostly orient parallel to the ED but the fine DRXed grains exhibit relatively random orientations. The area fractions of the DRXed grains in the FE and QE samples are measured to be 55% and 31%, respectively. The magnified SEM image inset given in Fig. 1b reveals the dense distribution of the nanoscale γ′ precipitates in the coarse unDRXed grains of the QE sample. These fine γ′ precipitates pin the dislocations effectively, then suppress the DRX process during extrusion process which leads to higher initial hardness of the QE sample. The average DRXed grain sizes in the FE and QE samples are about 1.5 μm and 1.1 μm, respectively.

### Age-hardening curves of the extruded alloys

**Figure 2:**
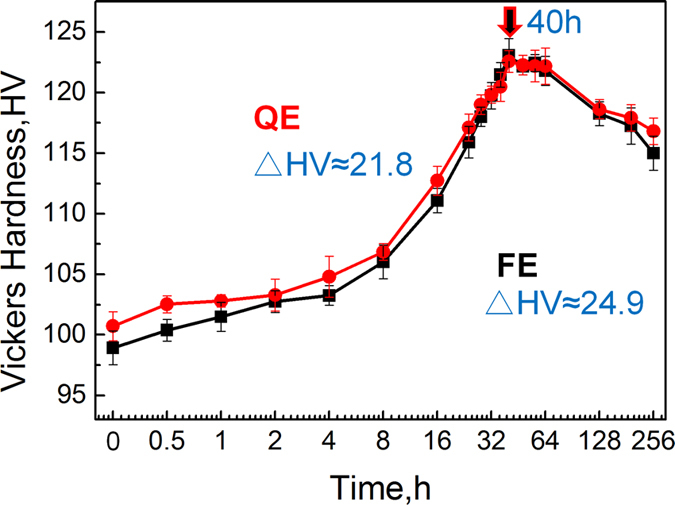
Age-hardening curves of the extruded alloys at 200 °C.

Figure 2 shows the age-hardening curves of the extruded alloys aged at 200 °C. It can be seen that both the FE and QE samples exhibit remarkable age-hardening response and similar ageing progress. The hardness of both alloys increases gradually at the early stage then rises rapidly after ageing for 8 h and reaches peak hardness at 40 h. After peak ageing, the hardness keeps stable until 64 h then decreases gradually. It is noted that the FE sample shows lower hardness before ageing treatment but shows almost same peak hardness of about 123 HV as the QE sample, in other words, the FE sample exhibits more obvious age-hardening response (∆HV = 24.9), as shown in Fig. 2.

### Microstructure of the as-aged alloys

**Figure 3:**
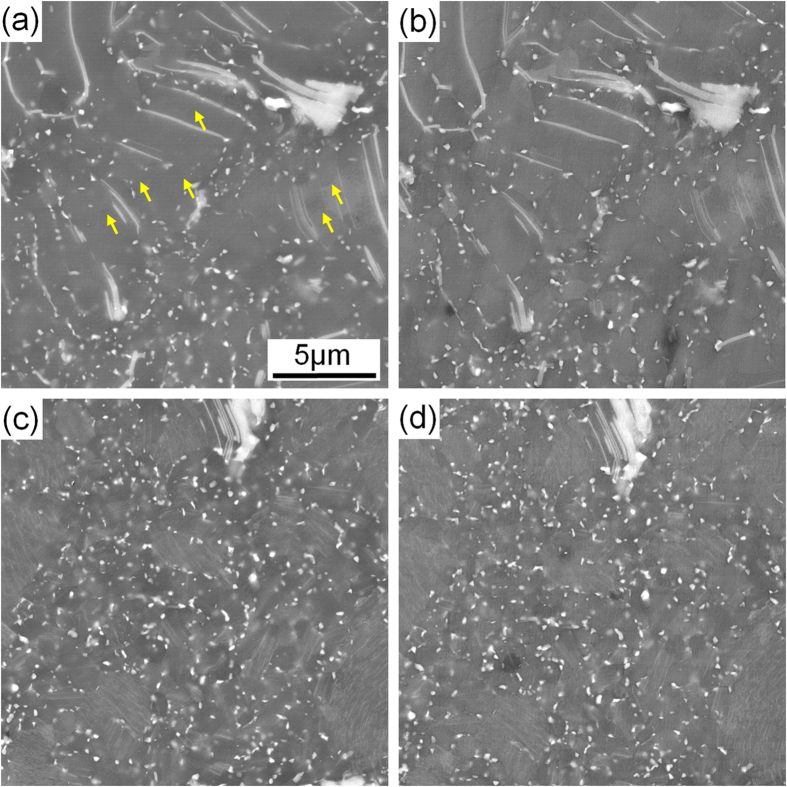
Quasi *in-situ* magnified SEM micrographs obtained from the regions marked by dotted boxes in Fig. 1: (**a**) FE, (**b**) FEA, (**c**) QE, (**d**) QEA.

**Figure 4:**
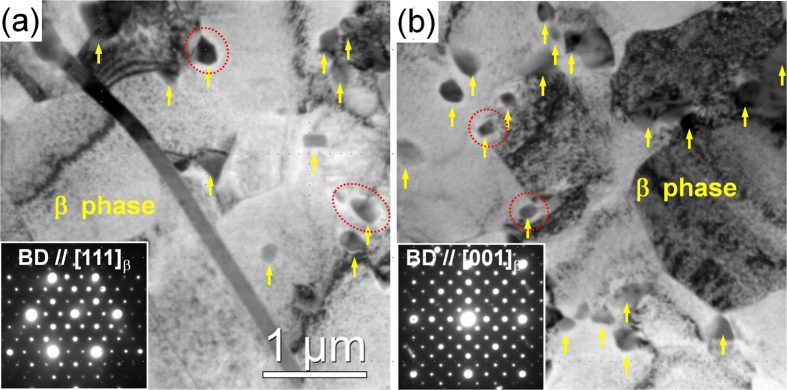
TEM BF images and corresponding SAED patterns taken from the DRXed regions of the as-extruded alloys: (**a**) FEA, (**b**) QEA.

Figure 1e and f show the SEM micrographs of the alloys after peak-ageing at 200 °C. It can be seen that the morphologies and sizes of the block and plate-shaped phases remain unchanged during ageing treatment by comparing with those in the extruded alloys (Fig. 1a and b). Additionally, quasi *in-situ* EBSD orientation maps demonstrate that no obvious grain growth occurs, and the grain orientations keep unchanged after the ageing treatment (comparison between Fig. 1c,d and g,h). This indicates that the microstructure of both alloys exhibits excellent thermal stability at 200 °C. Quasi *in-situ* SEM observations performed on the DRXed regions marked by yellow dotted boxes in Fig. 1 are shown in Fig. 3. Lots of nanoscale particles can be observed in both FE and QE samples (Fig. 3a and c) due to the dynamic precipitation during extrusion and majority of them distribute along the DRXed grain boundaries. There is negligible difference in the average diameters of the precipitates in both alloys, with the value of about 200 nm. After ageing treatment, no obvious particle growth or precipitation can be observed in the SEM images (Fig. 3b and d). Figure 4 shows the TEM bright field (BF) images and corresponding selected area electron diffraction (SAED) patterns of the dynamically precipitated particles in the DRXed regions of the peak-aged alloys. Fine β phase (Mg_5_Gd, fcc, F
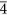
3 m, a = 2.23 nm)^19^ particles can be observed to distribute mainly at the DRXed grain boundaries. During the ageing treatment, these equilibrium β phases with good thermal stability effectively pin the DRXed grain boundaries and restrict the grain growth for both alloys, leading to high thermal stability of the microstructure. In addition, narrow precipitate free zones with width of about 20~30 nm can be observed around the β phases, as marked by red dotted circles in Fig. 4. The solute atoms and vacancies adjacent to the β phases diffuse to the interface during ageing treatment, leading to slight phase coarsening and the formation of precipitation free zones (PFZs) around the phases.

**Figure 5:**
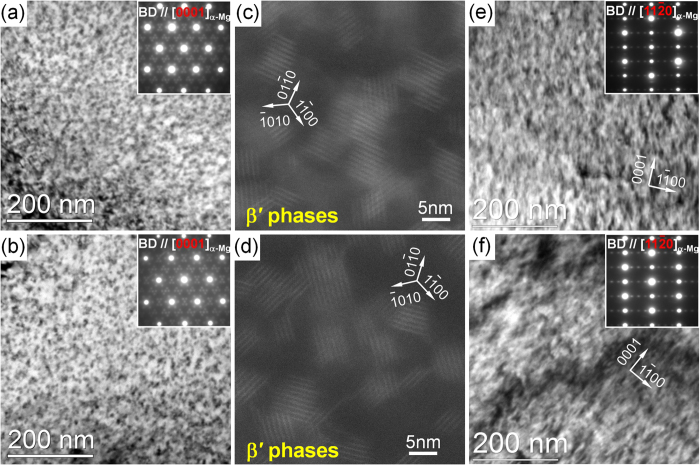
TEM and STEM observations on the DRXed grains of the peak-aged alloys: (**a**–**d**) BF images and STEM micrographs taken along [0001]_α-Mg_ direction, (**e**,**f**) BF images taken along [11
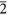
0]_α-Mg_ direction; (**a**,**c**,**e**) FEA, (**b**,**d**,**f**) QEA.

**Figure 6:**
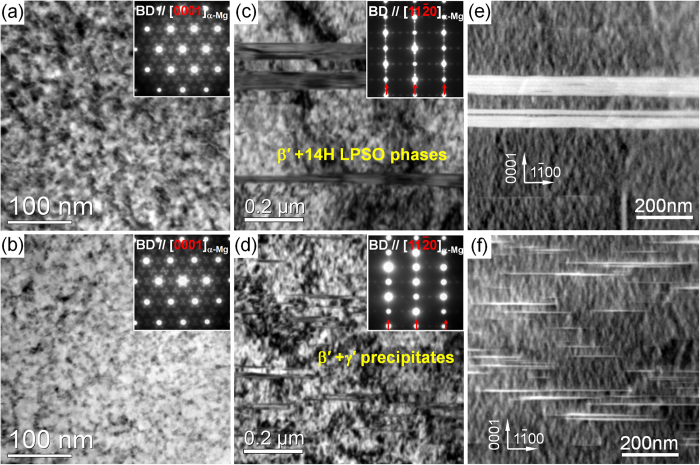
TEM and STEM observations on the unDRX grains of the peak-aged alloys: (**a**,**b**) BF images taken along [0001]_α-Mg_ direction, (**c**–**f**) BF images and STEM micrographs taken along [11
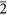
0]_α-Mg_ direction; (**a**,**c**,**e**) FEA, (**b**,**d**,**f**) QEA.

Figures 5 and 6 shows the TEM micrographs of the precipitates inside DRXed grains and unDRXed grains of the extruded alloys during peak-ageing treatment, respectively. The BF images and corresponding SAED patterns of the precipitates in the FE and QE samples taken along [0001]_α-Mg_ (Fig. 5a and b) indicate that dense nanoscale β′ phases with base-centered orthorhombic (bco) structure (a = 0.650 nm, b = 2.272 nm, c = 0.521 nm)^5^,^20^,^21^ form during ageing treatment. By using the convergent beam electron diffraction (CBED) technique^22^, the number density of them in the FEA and QEA samples are estimated to be about 7.2 × 10^22^ m^−3^ and 6.4 × 10^22^ m^−3^, respectively, which means that more β′ phases precipitate in the FEA sample. It is likely related to the fact that denser β phases dynamically precipitate during extrusion process (Fig. 3), which consumes more solute atoms inside the DRXed grains of the QE sample than that of the FE.

Based on the HAADF-STEM images obtained along [0001]_α-Mg_ directions (Fig. 5c and d), the morphologies of the β′ precipitates in both alloys are nearly equiaxed when observed along c-axis of α-Mg, that is, the aspect ratios (defined as precipitate length in the [010]_β′_//[10
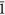
0]_α-Mg_ direction divided by length in the [100]_β′_//[11
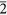
0]_α-Mg_ direction) of them are nearly 1, which is different from those with lenticular shape along [10
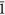
0]_α-Mg_ direction in the previously reported peak-aged Mg–Gd based alloys^20^,^21^,^23^,^24^. It is reported that the morphology of the β′ precipitates depends on the competition between the interfacial energy and elastic strain energy anisotropy. Additionally, the aspect ratio of the precipitates is more sensitive to the variation of the lattice parameters, i.e. the elastic strain energy^24^. The partition of Zn (radius of about 0.134 nm) with smaller atomic radius into the β′ precipitates may reduce the elastic strain energy anisotropy caused by the large size of Gd/Y atoms, thereby decreasing the aspect ratio of the phase to about 1. When observed along [11
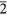
0]_α-Mg_ directions, the β′ precipitates have an elongated shape to the c axis//[0001]_α-Mg_ direction as indicated in the Fig. 5e and f, thus they show rod morphology along c axis. The average diameters of β′ precipitates on the basal planes in the both FEA and QEA samples are almost identical, namely as fine as about 7 nm. Therefore, the higher solute concentration in the DRXed grains of the FEA sample leads to denser nuclei than the QEA sample, but has negligible influence on size of the precipitates. Figure 6 shows the TEM observations of the precipitates in the unDRXed grains of the FEA and QEA samples. The β′ also precipitates inside the unDRXed grains and the number densities of them are estimated to be about 6.9 × 10^22^ m^−3^ and 6.1 × 10^22^ m^−3^ in the FEA and QEA samples, respectively (Fig. 6a and b), which are slightly lower than those inside the DRXed grains. The denser precipitation of the β′ precipitates in both DRXed and unDRXed regions of FEA accounts for its higher age-hardening response. The plate-shaped 14H LPSO phases and γ′ precipitates on the basal planes of the α-Mg matrix in the FEA and QEA are observed, as shown in Fig. 6c–f. The corresponding HAADF-STEM images (Fig. 6e and f) indicate the solute segregation in the γ′ precipitates by the white contrast, similar as that in the LPSO phases. Therefore, the formation of the LPSO phases and γ′ precipitates consumes the solute atoms saturated in the α-Mg matrix, thus the number density of the β′ precipitates in the unDRXed regions is lower than that in the DRXed regions of both alloys. It is observed that the distribution of the coarse plate-shaped LPSO phases in the FEA is sparser than that of the fine γ′ precipitates in the QEA. The number densities of the LPSO phases in the FEA and γ′ precipitates in the QEA are calculated to be 3.6 × 10^18^ m^−3^ and 3.9 × 10^20^ m^−3^, respectively.

### Texture evolution

**Figure 7:**
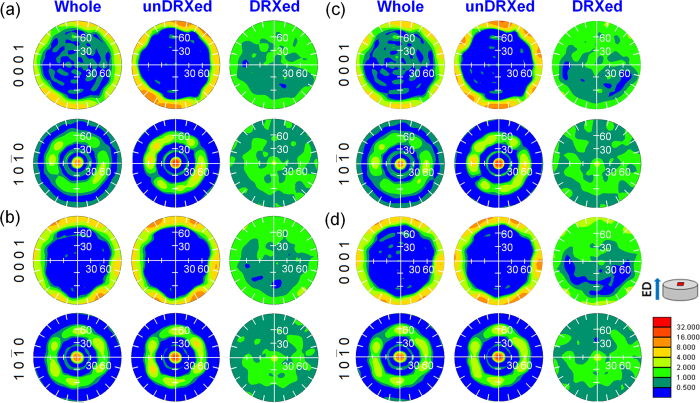
(0001) and (10
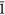
0) pole figures obtained from the different regions of the as-extruded and peak-aged samples: (**a**) FE, (**b**) QE, (**c**) FEA, (**d**) QEA.

Figure 7a and b show the (0001) and (10
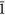
0) pole figures obtained from transverse sections of the extruded alloys by EBSD. It can be seen that both alloys exhibit the typical basal fiber texture with (0001) basal planes and <10
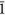
0> directions parallel to the ED^2^,^20^,^25^,^26^,^27^. In order to analyze the contribution of unDRXed and DRXed regions to the texture, the pole figures of the two regions are given in Fig. 7. The unDRXed regions show a strong basal fiber texture, while the DRXed regions have a much weaker basal texture due to their almost random orientations (Fig. 1). Furthermore, because the volume fraction of unDRXed grains in the QE sample is 22% higher than that in the FE sample (Fig. 1), the QE sample shows stronger basal fiber texture than the FE sample.

Quasi *in-situ* texture analysis of the alloys before and after ageing treatment was carried out and the pole figures of the peak-aged alloys are given in Fig. 7c and d. The texture remains unchanged after ageing treatment due to the excellent thermal stable in microstructure of the alloys during ageing at 200 °C.

### Mechanical properties

**Figure 8:**
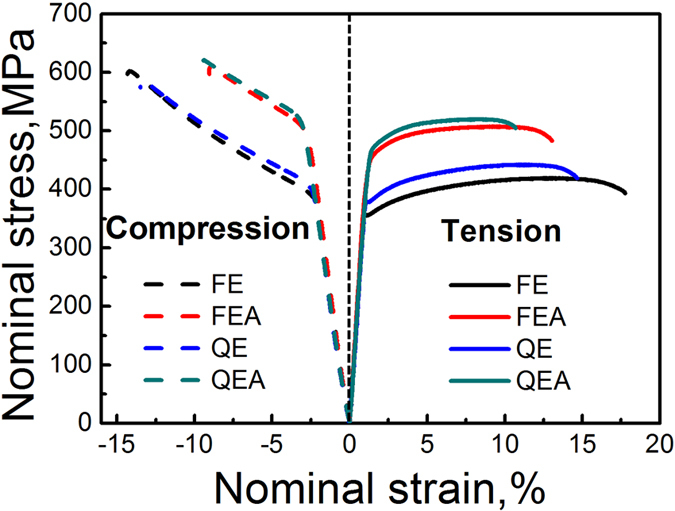
Tensile and compressive nominal stress-strain curves at RT.

Figure 8 shows the tensile and compressive stress-strain curves of the samples tested at RT and the corresponding ultimate tensile/compressive strength (σ_UTS_/σ_UCS_), tensile/compressive yield strength (σ_TYS_/σ_CYS_) and elongations to failure are summarized in Table 1. It can be seen that after T5 peak ageing at 200 °C, the FEA sample exhibits σ_TYS_ of 446 MPa, UTS of 508 MPa, and elongation to failure of 13.1%, while the QEA sample has σ_TYS_ of 462 MPa, UTS of 520 MPa, and elongation to failure of 10.6%. It is noted that the strength of both the FEA and QEA in this study are much higher than the previously reported Mg–Gd–Y–Zn–Zr alloys^28^,^29^,^30^,^31^,^32^ except for Mg–10.1Gd–5.7Y–1.6Zn–0.5Zr (wt.%) alloy^18^. The extruded and peak-aged Mg–10.1Gd–5.7Y–1.6Zn–0.5Zr (wt.%) alloy has σ_TYS_ of 473 MPa, σ_UTS_ of 542 MPa^20^, which are slightly higher than those obtained in the present study, while its elongation to failure is lower than that of the present alloy. It should be noted that the present alloy containing 12 wt.% RE, much lower than that of Mg–10.1Gd–5.7Y–1.6Zn–0.5Zr (wt.%), has superior cost performance and potential industrial applications.

The high strength of the peak-aged Mg–Gd based alloys is ascribed to solution strengthening, grain boundary strengthening, dislocation strengthening as well as precipitation strengthening induced by ageing treatment. The contribution of each strengthening mechanism to the yield strength of the present T5-treated alloy is discussed.

#### Solid solution strengthening

It is well known that the HRE elements have high solid solubility in α-Mg matrix which is about 0.61 at.% for both Gd and Y at ageing temperature of 200 °C^31^. Additionally, it is reported that Y is an effective solute strengthener for basal slip in Mg alloys due to its large solute misfits and high solubility^33^. Thus it can be deduced that Gd with similar atomic radius as Y and higher solubility shows higher potential for strengthening the basal slip, thus effective solution strengthening can be expected in both alloys in this study. While the equilibrium solid solubility of Gd and Y in α-Mg matrix of FEA and QEA at ageing temperature of 200 °C is identical. Accordingly, there may be negligible difference in solution strengthening between the FEA and QEA.

#### Grain boundary strengthening

The IPF maps (Fig. 1) reveals the fine DRXed grain size for both FEA and QEA alloys which contributes to the high strength of the alloys based on the Hall-Petch relationship and the increment in strength caused by grain boundaries in the DRXed regions (Δσ_GB,DRX_) can be given as follow:


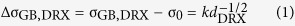


where σ_GB,DRX_ is the experimental yield strength, σ_0_ is the intrinsic strength of the single crystal without grain boundaries, *k* is the HP coefficient which is about 164 MPa μm^−1/2^ for extruded and peak-agd Mg–Gd–Y–Zr alloys^32^ an*d d*_DRX_ is the average DRXed grain size. Thus the Δσ_GB,DRX_ in the FEA and QEA are calculated to be 134 MPa and 156 MPa, respectively.

#### Dislocation strengthening

Color variations caused by the dense dislocations in the unDRXed grains of both alloys can be clearly observed in the IPF maps shown in Fig. 1. Therefore, dislocation strengthening (Δτ_d,unDRX_) caused by the high density of dislocations in the unDRXed regions can be calculated from the Eq. (2):


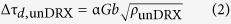


where α is a constant and is assumed to be 0.5^34^, *G* is the shear modulus of the α-Mg matrix (about 16.6 GPa^35^), *b* is the Burger vector (0.32 nm for Mg^35^) and *ρ*_unDRX_ is the dislocation density in the unDRX regions which is difficult to be measured accurately. However, the density of the geometrically necessary dislocations (GNDs) can be calculated by using the EBSD technology. Therefore, we can estimate the difference in the dislocation strengthening for FEA and QEA based on the calculation of GNDs density, despite of the existence of static dislocation. The average densities of GNDs in the unDRXed regions shown in Fig. 1g and h are calculated by CrossCourt3 software to be 2.5 × 10^14^ m^−2^ for the FEA and 2.8 × 10^14^ m^−2^ for the QEA, respectively, which are comparable to the dislocations density reported in the deformed Mg alloys^36^,^37^. Thus Δτ_d,unDRX_ in the FEA and QEA are estimated to be 42 MPa and 44 MPa, respectively. Since the grain boundary strengthening induced by coarse unDRX grains and dislocation strengthening induced by the DRXed grains with low dislocation density are negligible, we simply estimate the contribution of grain boundary strengthening from the fine DRXed grains (Δσ_GB_) and dislocation strengthening from the unDRXed grains (Δτ_*d*_) to σ_TYS_ weighted by the DRX ratios (*f*_DRX_), then we can get


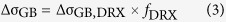


and


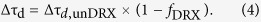


Therefore, Δσ_GB_ and Δτ_*d*_ are calculated to be 74 MPa and 19 MPa for the FEA as well as 49 MPa and 31 MPa for the QEA, respectively.

#### Precipitation strengthening

Since the dynamically precipitated β phases mainly distribute at the DRXed grain boundaries, their contribution to the yield strength is considered to be reflected by the grain boundary strengthening given in section 3.5.1. The yield strength at RT should be mainly associated with the basal dislocation slip and the increment in the critical resolved shear stress (CRSS) for basal slip is reported by Nie *et al*.^38^. The precipitation strengthening induced by basal plate-shaped LPSO phases 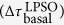 as well as γ′ precipitates 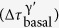 with large aspect ratios and the dense β′ precipitates with rod morphologies along the c-axis of α-Mg matrix 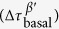 can be calculated by the same Eq. (5)^38^:


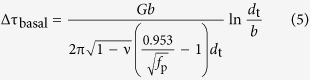


where ν is the Poisson’s ratio (ν = 0.32), *f*_*p*_ is the volume fraction of the precipitates and *d*_*t*_ is the uniform diameter of the precipitates. The volume fractions of rod β′ precipitate (*f*_*β*′_) and plate LPSO phase (*f*_LPSO_) as well as γ′ precipitate (*f*_γ_′) can be calculated by Eq. (6)^39^


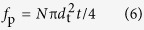


where *N* is the number density of the precipitates, *t* is the length/thickness of the precipitates, *d*_*t*_ and *t* of the precipitates are summarized in Table 2. Since the LPSO phases and γ′ precipitates mainly distribute in the unDRXed regions and the number densities of the β′ precipitates in the DRXed and unDRXed regions are different, the contributions of precipitation strengthening from DRXed and unDRXed regions are calculated separately. 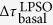 and 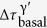 in the unDRXed regions for the FEA and QEA are calculated to be 1.6 MPa and 11 MPa, respectively, suggesting that the finer γ′ precipitates exhibits much higher strengthening effect than the coarse LPSO phases. 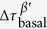 in the DRXed and unDRXed regions are calculated to be 167 MPa and 164 MPa for the FEA as well as 164 MPa and 160 MPa for the QEA, respectively. Therefore, the strengthening caused by the β′ precipitates is slightly higher in the FEA than QEA and prismatic precipitates are much effective than the LPSO phases and γ′ precipitates in blocking the basal dislocation slip.

**Figure 9:**
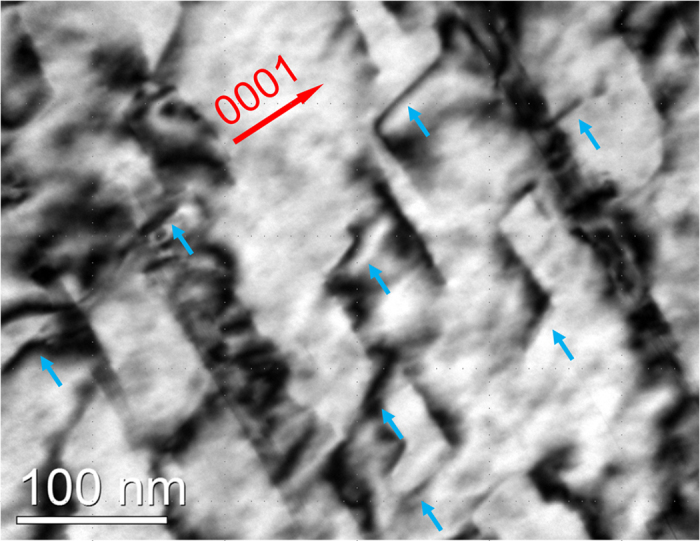
TEM BF image taken from the unDRXed grain of QEA after tensile test at RT with strain of 5% (B = [11
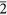
0], g = (0002)).

Figure 1i and j show the (0001) <11
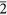
0> Schmid factor distribution maps of the peak-aged alloys, which indicates that DRXed regions have high Schmid factors but unDRXed regions have lower Schmid factors. As a result, basal slip in the unDRXed regions is difficult to be activated during tensile test along ED at RT. It is reported that Y addition increases CRSS of basal slip more than that of second-order pyramidal <c + a> slip ({11
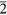
2} <11
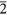
3>), which reduce the difference in CRSS between them, therefore, activation of <c + a> slip was observed in Mg–Y alloys at RT^40^. Gd also satisfies the conditions for increasing the activity of non-basal (particular <c + a> dislocations) slip at RT^41^. Figure 9 shows the TEM BF image of the dislocation substructures developed in unDRXed grains of QEA after tensile test to 5% strain, the image was taken using a zone axis of [11
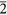
0] with g = (0002). Lots of <c + a> dislocations are observed to be bounded by the basal γ′ precipitates, as indicated by blue arrow heads, and thus, in present study, <c + a> slip is demonstrated to be activated in the unDRXed regions where the activity of the basal slip is obviously impeded during the tensile test at RT. The activity of the <c + a> dislocation slip affects the flow stress and hardening rates so that it is necessary to evaluate the precipitation strengthening on the <c + a> slip. Recently, Wang *et al*.^42^ suggested an Orowan equations for predicting the strengthening of non-basal slip systems in hexagonal crystals for precipitates with typical morphologies and orientations. In the present study, the contribution of LPSO phases, γ′ precipitates with basal-plate morphologies (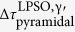) and β′ precipitates with [0001] rod morphology (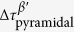) in the unDRXed regions to the strengthening of {11
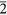
2} <11
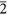
3> second-order <c + a> slip are calculated as follows^43^:


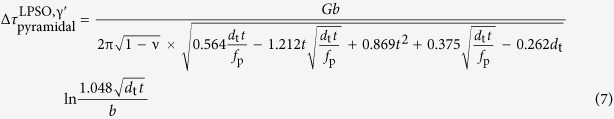



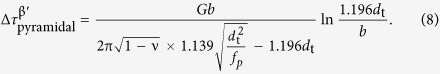


Based on the parameters given above, 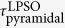 for the FEA and 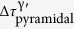 for the QEA are calculated to be 11.6 MPa and 52 MPa, respectively, which are much higher than 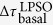 and 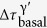. It means that basal LPSO phases and γ′ precipitates are weak strengthener for basal slip, while they block pyramidal <c + a> dislocations more effectively. 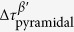 in the DRXed and unDRXed regions are estimated to be 155 MPa and 150 MPa for the FEA as well as 150 MPa and 146 MPa for the QEA, respectively, which are slightly lower than those for strengthening the basal slip.

The calculations reveal that contribution of the precipitation strengthening induced by the β′ precipitates is much higher than the other strengthening factors. The precipitation of γ′ precipitates leads to lower number density of β′ precipitates in the QEA, so that β′ precipitates bring about lower increment in strength for the QEA than FEA. However, based on the abovementioned calculation, the γ′ precipitates in the unDRX regions of the QEA are more effective in strengthening both basal and non-basal slip than the LPSO phases in the FEA, and the volume fraction of the unDRXed regions is higher in the QEA. As a result, the QEA shows higher tensile yield strength than the FEA.

The yield anisotropies of the extruded and peak-aged alloys defined by CYS/TYS are summarized in Table 1. Due to the activation of {101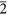} tension twinning during compression test along ED, strong yield anisotropy with lower yield strength in compression was widely reported in the extruded commercial Mg alloys such as AZ61^44^, while it is not observed in this study. Kula *et al*.^45^ reported that the addition of 0.3 at.% Gd and 0.5 at.% Y and into Mg, facilitate higher activity of slip than twinning during compression deformation at RT, which results to the drastically reduced yield anisotropy of 0.83 and 0.77 for Mg–Gd and Mg–Y alloys, respectively (0.21 for pure Mg). It is noted that the equilibrium solid solubility of Gd/Y in α-Mg matrix at 200 °C is about 0.61 at.%, thus much lower yield anisotropy can be expected. The precipitation of the basal LPSO phases and dense γ′ precipitates can effectively inhibit twinning during compression along ED. As a result, dislocation slip dominates the both tensile and compressive deformation at RT, leading to equal yield strength in tension and compression, that is, yield anisotropy should be 1. However, it is contrary to the experimental result that inverse phenomenon with higher strength in compression than in tension is obtained for both extruded and peak-aged alloys in this study. Garces *et al*.^46^ also observed such reversed yield anisotropy in a LPSO phase containing Mg-Y-Zn alloys and ascribed this phenomenon to the dislocation slip dominated in DRX regions with area fraction higher than 50% and large amount of LPSO phase which is stronger in compression than in tension. While the QE and QEA with low DRX ratio of 31% in the present study also show reversed yield anisotropy, the values are almost identical to those values for the FE and FEA with plate-shaped LPSO phases. This indicates that the dense γ′ precipitates may also show higher strength in compression then in tension as LPSO phases and further research is needed to clarify the mechanism.

In summary, the effect of the long period stacking ordered (LPSO) phases and γ′ precipitates on the ageing behavior and mechanical properties of extruded Mg–8.2Gd–3.8Y–1Zn–0.4Zr alloy was investigated, we can conclude that the LPSO phase containing alloy exhibit higher age-hardening response than the γ′ precipitates containing alloy, which is due to the slightly higher number density of β′ phases precipitated during ageing treatment in the LPSO phase containing alloy than those in the γ′ precipitates containing alloy. High strength and moderate ductility are obtained in both peak-age alloys. Based on the calculations, the precipitation strengthening induced by the β′ precipitates plays the most important role in the high strength of the alloy. Although the β′ precipitates contribute more to the strength of the LPSO phase containing alloy, the γ′ precipitates containing alloy show higher strength due to the more effective strengthening induced by dense nanoscale γ′ precipitates than LPSO phases as well as the higher volume fraction of the unDRXed grains. The peak-aged γ′ precipitates containing alloy exhibits tensile yield strength of 462 MPa, ultimate tensile strength of 520 MPa and elongation to failure of 10.6%.

## Methods

### Material preparation

Mg–8.2Gd–3.8Y–1.0Zn–0.4Zr (wt.%) alloy ingot with 280 mm in diameter and 2940 mm in length was produced by direct chill casting^16^. The specimens machined from the ingot were homogenized at 510 °C for 12 h in a Pyrex tube under an Ar atmosphere, then cooled in furnace to ambient temperature with speed of ~0.7 °C/min. While some other samples were immediately quenched in warm water of about 80 °C after homogenizing treatment at 510 °C for 12 h in a Pyrex tube under an Ar atmosphere. The homogenized samples were machined to cylindrical samples with 43 mm in diameter and 50 mm in height for extrusion, then preheated at 400 °C for 5 min prior to the extrusion for homogenizing the temperature of the samples. The extrusion rods with 13.6 mm in diameter and 400 mm in length was produced by indirect extrusion at 400 °C with an extrusion ratio of 10:1 and a ram speed of 0.1 mm/s. The extrusion rods from furnace-cooled alloy and quenched alloy are denoted as FE and QE, respectively. The extruded alloys were then subjected to ageing treatment at 200 °C. The peak-aged alloys are denoted as FEA and QEA, respectively.

### Microstructure characterization

In order to analyze the microstructure and texture evolution of the extruded alloys during ageing treatment, quasi *in-situ* SEM observation and EBSD analysis were performed using JEOL JSM-7000F field-emission scanning electron microscope (FE-SEM) equipped with an EDAX-TSL EBSD system operating at 25 kV, and the data were analyzed by OIM Analysis software. Firstly, the SEM micrographs and EBSD scans were taken from cross-section of the extruded alloys. Secondly, the extruded alloys were peak-ageing treated in oil bath and then slightly mechanically polished using alumina suspension to remove the oxidized layer. Finally, the same positions as those on the extruded alloys were observed by SEM and EBSD. The precipitation behavior of the peak-aged alloys was analyzed by JEOL JEM-2100F Transmission electron microscope (TEM) and high angle annular dark field scanning transmission electron microscopy (HAADF-STEM) operating at 200 kV. Thin foils for TEM observation with thickness of 0.2 mm were punched into discs with 3 mm in diameter and mechanically polished followed by low angle ion milling using Gatan precision ion polishing system.

### Mechanical property tests

Hardness was measured by the VMT-7S Vickers hardness testing machine with a load of 4.9 N and a loading time of 15 s. The tensile specimens having a gauge length of 30 mm and a diameter of 6 mm and compressive specimens with a length of 15 mm and a diameter of 6 mm were machined from the as-extruded rods. The tensile and compressive tests with tensile and compressive directions parallel to the extrusion direction (ED) were conducted on a Shimadzu Autograph AG-I (50 kN) machine at an initial strain rate of 1 × 10^−3^ s^−1^ at room temperature (RT).

## Additional Information

**How to cite this article:** Xu, C. *et al*. Ageing behavior of extruded Mg–8.2Gd–3.8Y–1.0Zn–0.4Zr (wt.%) alloy containing LPSO phase and γ′precipitates. *Sci. Rep.* **7**, 43391; doi: 10.1038/srep43391 (2017).

**Publisher's note:** Springer Nature remains neutral with regard to jurisdictional claims in published maps and institutional affiliations.

## Figures and Tables

**Table 1:** Tensile and compressive properties of the samples tested at RT.

	TYS, MPa	UTS, MPa	ε, %	CYS, MPa	UCS, MPa	ε, %	CYS/TYS
FE^26^	356	419	17.8	380	602	14.3	1.07
FEA	446	508	13.1	490	610	9.0	1.09
QE^26^	379	442	14.7	401	586	13.4	1.06
QEA	462	520	10.6	520	621	9.5	1.12

**Table 2:** Parameters of the precipitates for the strengthening calculations.

	FEA		QEA	
	LPSO	β′	γ′	β′
*d*_t_ (nm)	680	7	190	7
*t* (nm)	48	25	5	26
